# The *rem* Mutations in the ATP-Binding Groove of the Rad3/XPD Helicase Lead to *Xeroderma pigmentosum*-Cockayne Syndrome-Like Phenotypes

**DOI:** 10.1371/journal.pgen.1004859

**Published:** 2014-12-11

**Authors:** Emilia Herrera-Moyano, María Moriel-Carretero, Beth A. Montelone, Andrés Aguilera

**Affiliations:** 1 Centro Andaluz de Biología Molecular y Medicina Regenerativa CABIMER, Universidad de Sevilla, Seville, Spain; 2 Division of Biology and College of Arts & Sciences, Kansas State University, Manhattan, Kansas, United States of America; Duke University, United States of America

## Abstract

The eukaryotic TFIIH complex is involved in Nucleotide Excision Repair and transcription initiation. We analyzed three yeast mutations of the Rad3/XPD helicase of TFIIH known as *rem* (recombination and mutation phenotypes). We found that, in these mutants, incomplete NER reactions lead to replication fork breaking and the subsequent engagement of the homologous recombination machinery to restore them. Nevertheless, the penetrance varies among mutants, giving rise to a phenotype gradient. Interestingly, the mutations analyzed reside at the ATP-binding groove of Rad3 and *in vivo* experiments reveal a gain of DNA affinity upon damage of the mutant Rad3 proteins. Since mutations at the ATP-binding groove of XPD in humans are present in the *Xeroderma pigmentosum*-Cockayne Syndrome (XP-CS), we recreated *rem* mutations in human cells, and found that these are XP-CS-like. We propose that the balance between the loss of helicase activity and the gain of DNA affinity controls the capacity of TFIIH to open DNA during NER, and its persistence at both DNA lesions and promoters. This conditions NER efficiency and transcription resumption after damage, which in human cells would explain the XP-CS phenotype, opening new perspectives to understand the molecular basis of the role of XPD in human disease.

## Author Summary

TFIIH is a protein complex that functions in the repair of bulky adducts distorting the DNA via the pathway of Nucleotide Excision Repair, and in transcription initiation and transactivation, the latter being a specific transcription activation process occurring in response to hormones. We have taken advantage of the powerful genetics and molecular biology of the model organism *Saccharomyces cerevisiae* to characterize the impact on cell fitness of a particular kind of mutations of one of the two helicases of the TFIIH complex, Rad3, called *rem* mutations for their increased levels of recombination and mutation. We have realized that these mutations affect a particular site of the protein, its ATP-binding groove, and modify the dynamics of TFIIH, leading to unfinished repair reactions and DNA break accumulation. Finally, we recreated these mutations in the human homolog XPD protein and found that their phenotypes recapitulated those of human mutations leading to a combination of the two hereditary diseases *Xeroderma pigmentosum* and Cockayne syndrome (XP-D/CS), whose molecular basis remains elusive. As these mutations also affect the ATP-binding groove of XPD, this study permits to propose a model to explain the molecular basis of XP-D/CS.

## Introduction

Accuracy of DNA enzymatic processes, such as transcription, replication and repair, is essential to guarantee genome integrity and, at a higher scale, cell and organism fitness. Such processes are functionally connected to checkpoint mechanisms that respond to DNA damage and stresses compromising cell cycle progression [1]. One relevant player in DNA repair and the maintenance of genome integrity is the multifunctional eukaryotic complex TFIIH. It is formed by 10 subunits and functions in Nucleotide Excision Repair (NER), transcription initiation and transactivation. During NER, bulky adducts that distort the DNA helix are recognized as lesions to which TFIIH binds to allow DNA unwinding, damaged DNA strand recognition and recruitment of the specific nucleases that excise the damaged DNA segment. During transcription, TFIIH facilitates DNA strand opening at promoter regions allowing full association of the transcription machinery and transcription initiation. Promoter escape, which allows transition from transcription initiation to elongation, is achieved by the ability of the cAMP-kinase CAK subcomplex of TFIIH to phosphorylate the C-terminal domain of RNA polymerase II (RNAPII) [2], [3]. During transactivation, TFIIH phosphorylates nuclear receptors to allow their entry into the nucleus, which in turn activates expression of downstream genes.

Central to TFIIH performance is Rad3/XPD (as named in yeast/mammals), an essential and conserved eukaryotic protein with 5′>3′ DNA helicase activity. During NER, Rad3 catalyzes DNA-strand opening. This creates the substrate for the action of the DNA-incision endonucleases Rad1-10/XPF-ERCC1 and Rad2/XPG. It is believed that removal of TFIIH is required to allow re-filling of the ssDNA gap generated by the endonucleases [4]. In contrast, the role of Rad3 in transcription initiation is structural. The activity required to open the promoter is provided by a second helicase present in TFIIH, Rad25/XPB [5]. Rad3 serves as a bridge between the core TFIIH and the CAK subcomplex. Since, as mentioned above, CAK phosphorylates RNAPII to clear the promoter and is responsible for the phosphorylation of nuclear receptors during transactivation, Rad3 integrity is fundamental for CAK attachment to TFIIH and its correct performance during transcription and transactivation.

Altogether, this explains why mutations in *XPD/RAD3* may lead to NER failures as well as transcriptional and developmental defects. In humans, *XPD* mutations lead to *Xeroderma pigmentosum* (XP) and trichothiodystrophy (TTD), as well as combinations of XP with Cockayne Syndrome (XP-CS) and with TTD (XP-TTD). The clinical features of XP patients are explained by a NER deficiency, while TTD is seen as a consequence of transcriptional defects. However, our understanding of the XP-CS clinical features is less clear [6]. CS phenotypes are attributed to the inability to perform transcription-coupled repair (TCR), a NER subpathway in which lesions in the transcribed DNA strand encountered by an elongating RNAPII are efficiently repaired as compared to those of the non-transcribed strand [7], [8]. In a simplified way, XP-D/CS patient phenotypes could thus be explained by a TCR defect and/or a transcription defect, which would also explain their developmental defects and extreme sunlight sensitivity. Nevertheless, additional issues complicate this view: DNA breaks happen *in trans* upon UV irradiation in XP-D/CS cells [9] and the cancer proneness of XP-D/CS mice exceeds that of the most NER-deficient mice, those lacking XPA [10]. Therefore, there is still a lack of comprehension regarding the molecular basis of XPD-associated XP-CS phenotypes.

A particularly interesting class of *rad3* mutations in *Saccharomyces cerevisiae* is that comprising the semi-dominant *rad3-101* (Rad3-A237T) and *rad3-102* (Rad3-H661Y) alleles [11]. They were named *rem* alleles, as they displayed increased levels of *re*combination and *m*utation [12], [13]. *rem* mutants differ from canonical NER-deficient *RAD3/XPD* mutants in their moderate UV sensitivity, increased levels of allelic recombination in heterozygous diploid cells, and inviability in the absence of the homologous recombination (HR) factor Rad52 [13], [14]. We have previously shown that, in contrast to most NER-deficient mutations, the *rad3-102* (Rad3-H661Y) allele blocks NER at a post-incision step causing an extended retention of TFIIH at the damaged DNA that in turn provokes replication fork breakage and channeling of bulky lesions into HR-mediated Double Strand Break (DSB) repair [15]. The longer stay of Rad3-H661Y-containing TFIIH complexes at the site of NER action may be explained by an elevated affinity for ssDNA, given that the *rad3-102* mutation lies in the ATP-binding groove of Rad3 and, when ATP hydrolysis by TFIIH is prevented, a gain of affinity for ssDNA manifests [16]. We have proposed that a parallelism may exist between *rad3-102* cells and the mutations causing XP-CS in humans [15] because of several reasons. First, re-creation in *Sulfolobus acidocaldarius* of the equivalent human XPD-G675R mutant protein, associated with XP-CS disease, lies at the ATP-binding groove and displays a ssDNA-binding affinity 164% above that of the WT [17]. Second, this same mutation causes DNA breaks upon UV irradiation [9], reminiscent of *rad3-102* cells. Third, as in *rad3-102*, removal of early NER proteins, such as XPA in mice, suppresses the phenotype of break accumulation conferred by the XP-D/CS mutation *XPD-G602D* [14], [18].

To gain insight into the molecular nature of the *rem* mutations and the possibility that they could relate to specific defects such as XP-CS, we analyzed two other yeast *rem* mutants, *rad3-101* [12], [14] and the new and uncharacterized *rad3-107* (Rad3-E236G; this paper). Both mutations also lie in the ATP-binding groove of the protein and, relevantly, all above-mentioned mutations are perfectly conserved between *S. cerevisiae* and humans (S1 Figure). We first determined the levels of NER deficiency and the dependency on HR of these two *rem* mutants, establishing a gradient of phenotypes. *In vivo* experiments show that when the Rad3 ATP-binding groove is mutated, TFIIH gains a higher affinity for DNA. Since mutations in the ATP-binding groove of *Sulfolobus acidocaldarius* XPD may provoke both a loss of helicase activity and a gain of ssDNA affinity [17], our results suggest that the balance between these two effects determines the ability of TFIIH to open DNA during NER and its recruitment to both DNA lesions and promoters. This would impair transcription resumption after DNA damage and NER, consistent with the CS phenotype. Last, we extended our study to human cells and show that the mutation equivalent to yeast *rad3-102* in human cell lines recapitulates the XP-D/CS phenotypes, opening a parallelism between *S. cerevisiae rem* alleles and XP-D/CS mutations.

## Results

### *RAD3 rem* mutants display a gradient of responses to UV irradiation

**Figure 1:**
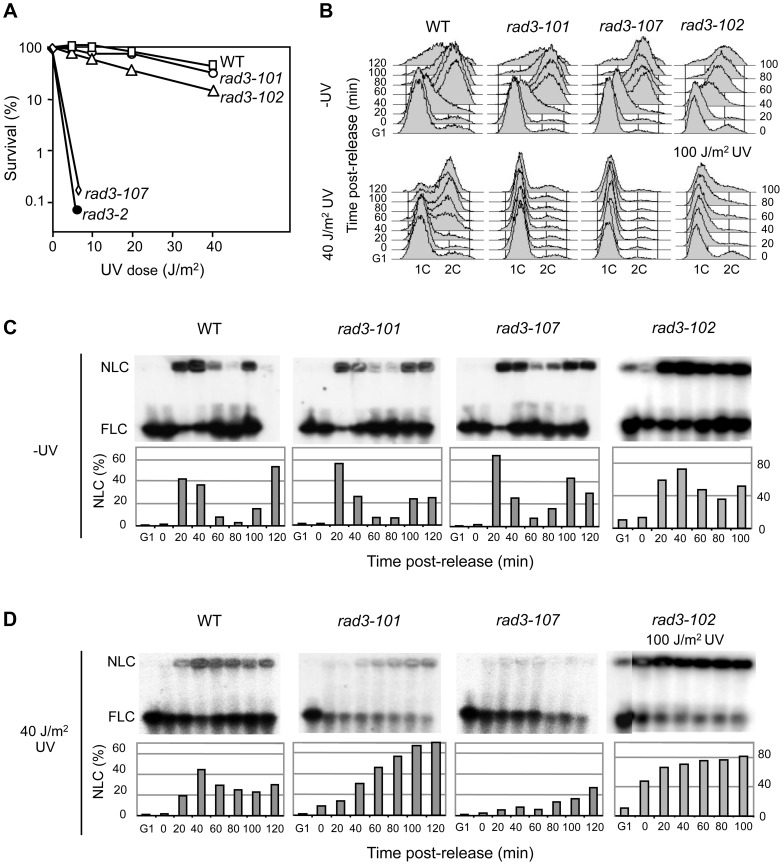
*rad3* mutants display a gradient response to UV irradiation. (**A**) Survival curves of WT and different yeast *rad3* mutants after UV-C exposure. (**B**) FACS profiles from WT, *rad3-101*, *rad3-107* and *rad3-102* cells synchronized in G1 with α-factor, untreated or UV-irradiated with 40 or 100 J/m^2^ and released after 2 h. (**C**) Pulsed-field gel electrophoresis (PFGE) of DNA from WT, *rad3-101*, *rad3-107* and *rad3-102* cells synchronized in G1 with α-factor and further released into S phase. Bands reveal chromosome VII by hybridization with a probe of the *ADE5,7 locus*. Nonlinear (NLC) and full-length linear (FLC) chromosomes include replication intermediates, in the well, and pre- and post-replicated chromosomes, which enter the gel, respectively. Bars represent the quantification of NLC with respect to the total of signal of each lane. The bottom part of the membrane below the FLC is not shown since no signals, as expected from broken DNA molecules, were revealed by hybridization in any lane. (**D**) All details as in (C) except that after G1 synchronization, cells were UV-irradiated with 40 J/m^2^ or 100 J/m^2^ and released into S phase 2 h later.

To define the molecular basis of the different phenotypes of *rad3-101* and *rad3-107*, we first studied the UV response of *rad3-101* and *rad3-107* mutants in comparison with that of the WT strain and the NER-deficient mutant *rad3-2*. Notably, *rad3-101* cells respond to UV as the WT strain and in contrast to *rad3-102* cells, which were slightly UV sensitive to increasing doses of UV. Instead *rad3-107*, as *rad3-2*, was highly UV-sensitive (Fig. 1A).

We have reported that *rad3-102* was moderately resistant to UV because DNA gaps generated by an unfinished NER reaction could be resolved during the S phase via recombination [15]. It is thus possible that UV lesions are differently processed in each of the *rad3* mutants analyzed, which would explain the different degrees of UV sensitivity. To test this possibility, we analyzed cell cycle progression through the S phase of cells synchronized in G1 with α-factor, irradiated with a 40 J/m^2^ UV-C dose and released 2 hours later from the G1 arrest. Without UV irradiation none of the mutant strains showed a cell cycle delay. However, while *rad3-102* cells were able to progress through S phase almost as readily as the WT after a similar UV dose [15], UV-treated *rad3-101* and *rad3-107* cells were not able to progress throughout the S phase (Fig. 1B). Since FACS analysis cannot differentiate between a block in G1 or early S phase, next we analyzed replication fork progression by PFGE. This technique allows us to determine the fraction of chromosomes that are under active replication as the fraction of DNA unable to enter the gel, staying stacked in the gel well during electrophoresis [15]. In agreement with the FACS analysis, replication kinetics was mostly unchanged without irradiation in the different assayed strains (Fig. 1C). When UV-irradiating the cells, the analysis reveals that *rad3-101* cells are able to initiate replication. Up to 70% of the DNA molecules were stacked in the well 120 min after G1 release (Fig. 1D). Nevertheless, replication in *rad3-101* was much slower than in the WT as it took longer to accumulate replicating chromosomes. Instead, the same UV dose seems to fully prevent the *rad3-107* UV-sensitive cells from initiating replication. In this case accumulation of replicating chromosomes in the well was poor (Fig. 1D). This *rad3-107* phenotype is reminiscent of that of the canonical NER-deficient mutant *rad3-2*, which is unable to progress into S phase after UV irradiation [15]. Therefore, the different replication efficiencies seem to match the distinct abilities of TFIIH to resolve DNA lesions via NER. According to this hypothesis, we would expect that, at higher UV doses, the UV-resistant *rad3-102* mutant should show a similar S-phase delay to that of the *rad3-101* and *rad3-107*. On the contrary, no arrest during the S phase would be expected for *rad3-101* cells at lower UV doses. Indeed, PFGE analysis showed a strong DNA retention in the well in *rad3-102* cells in early S phase at UV doses of 100 J/m^2^, reaching up to 80% of DNA molecules (Fig. 1B and D). Instead, after a 20 J/m^2^ UV dose, *rad3-101* and *rad3-102* cells entered S phase and progressed normally throughout the cell cycle, while *rad3-107* cells were still incapable to do so (S2 Figure). Altogether, these results indicate that NER is differently affected in the three mutants analyzed, the intensity of the defect correlating with a different degree of replication impairment.

### Recombinational repair in *rem* mutants

**Figure 2:**
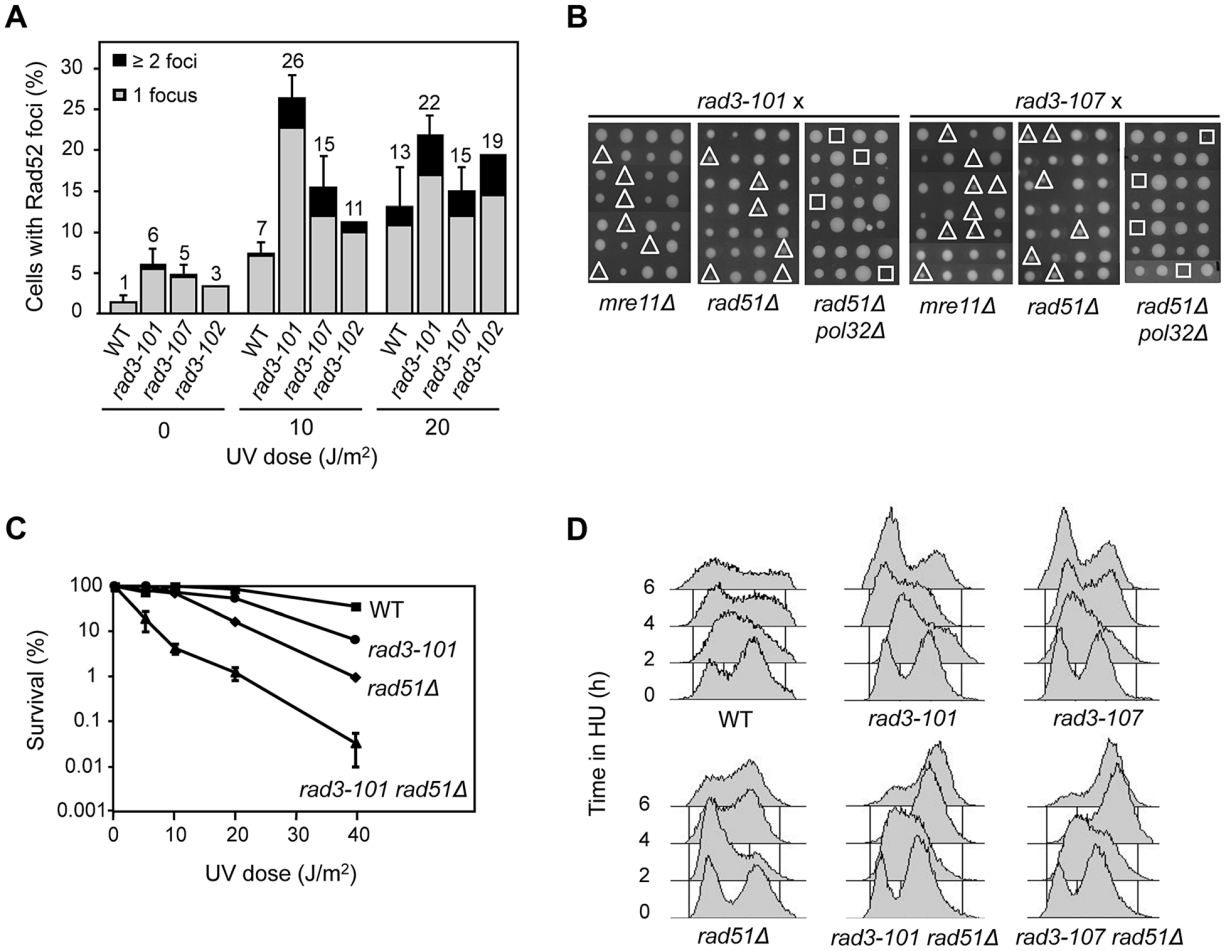
Hyper-recombination and genetic interactions of *rem* mutations with recombination and replication functions. (**A**) Analysis of Rad52 foci after exposure to UV-C. Only cells in S and G2 were considered. Error bars represent the SD of three independent experiments. One representative experiment is shown for *rad3-102* in order to facilitate comparison with previous published data [15]. (**B**) Analysis of genetic interactions of *rem* alleles of *RAD3* with *mre11Δ*, *rad51Δ* and *pol32Δ*. Tetrads dissected on rich medium are shown. The sites of double and triple mutants are indicated by triangles and squares, respectively. (**C**) Survival curves of WT, *rad3-101*, *rad51Δ* and their corresponding double mutant cells after UV-C exposure. (**D**) FACS profiles of mid-log cultures from WT, *rad3-101*, *rad51Δ* and their corresponding double mutant cells taken every 2 h after addition of 40 mM HU.

The spontaneous and UV-induced hyper-recombination of *rad3-102* mutants has been explained by the accumulation of ssDNA gaps derived from NER abortive reactions that are converted by replication into DSBs that are repaired by HR [15]. If this is the case for all *rem*-like mutants, the different amounts of DNA lesions accumulated in S phase as a consequence of abortive NER would be revealed by the levels of recombination. As genetically scored recombination events only inform about the DNA breaks that are successfully repaired by HR, we analyzed the global accumulation of Rad52 recombination foci as a way to evaluate whether a correlation existed between UV sensitivity and the accumulation of HR intermediates. Spontaneous and UV-induced Rad52 foci at 2 hours after irradiation with 10 or 20 J/m^2^ UV-C were thus analyzed and only cells in S and G2 phase of the cell cycle were considered. Both *rad3-101* and *rad3-107* cells accumulated more spontaneous Rad52 foci than wild-type cells (Fig. 2A), as previously shown for *rad3-102* ([15] and Fig. 2A). However, after 10 J/m^2^ of UV-C dose, *rad3-101* and *rad3-107* displayed a 4- and 2-fold increase with respect to the wild type, respectively (Fig. 2A), both mutants having reached a maximum level of Rad52 foci. After a 20 J/m^2^ UV-C dose, foci increased only in the wild type (Fig. 2A), consistent with the data showing that *rad3-101* and *rad3-107* cells did not progress through S phase properly (Fig. 1B). Notably, Rad52 foci accumulated in *rad3-107* despite its strong UV sensitivity, which contrasts with the canonical NER-deficient mutant *rad3-2* [15]. Altogether, these results suggest that a specific feature of *rem* mutants is their ability to convert bulky DNA adducts into DNA breaks that are repaired by HR, even though this may not be equally efficient in all mutants.

Consequently, we wondered whether early recombinational DSB repair functions such as Rad52 and the MRX complex become essential in *rad3-101* and *rad3-107* mutants, as previously shown for *rad3-102* and in contrast to *rad3-2* [13]-[15]. First we asked whether *rad3-101* and *rad3-107* relied on HR for their viability. *rad3-101* was either lethal or showed a synthetic growth defect phenotype when combined with *mre11Δ* in non-irradiated cells (Fig. 2B), consistent with the previous lethality reported for *rad3-101 rad52-1* [13]. The results suggest that, in *rad3-101*, spontaneous and UV-induced DNA breaks accumulate at a high frequency. *rad3-107* also proved to be dependent on HR functions for viability, as *rad3-107 mre11Δ* double mutants showed a clear synthetic growth defect (Fig. 2B). Moreover, surviving *rad3-101 mre11Δ* cells and *rad3-107 mre11Δ* cells were extremely UV-sensitive (S3 Figure). Data are consistent with the idea that a specific feature of *rem* mutants is the accumulation of DNA breaks that need to be repaired by HR, which thus becomes essential for viability, even though to different extents in different mutants.

Our previous findings that *rad3-102* is not viable if both Rad51 and Pol32 are removed suggest that these proteins control two different but non-mutually exclusive Rad52-dependent HR pathways for the repair of replication-mediated DNA breaks [15]. To assay whether this was also the case for the DNA lesions accumulated in *rad3-101* and *rad3-107* mutants, we characterized the corresponding double and triple mutants with *rad51Δ* and *pol32Δ*. As previously shown for *rad3-102* [15], double *rad3-101 rad51Δ* and *rad3-107 rad51Δ* mutants were viable. In addition, *rad3-101 rad51Δ* was clearly UV sensitive when compared with each of the single mutants (Fig. 2C). As can be seen in Fig. 2B, both triple mutants *rad3-101 rad51Δ pol32Δ* and *rad3-107 rad51Δ pol32Δ* were not viable, as previously reported for *rad3-102.* To assay whether Rad51 becomes critical for the repair of broken forks and viability under HU-induced replicative stress, we deleted it in *rad3-101* and *rad3-107* cells. Notably, when 40 mM HU was added to asynchronous cultures of both *rad3-101 rad51Δ* and *rad3-107 rad51Δ* double mutants, cells arrested in late S/G2 phase, in contrast to WT and single *rad3* mutants (Fig. 2D), as previously shown for *rad3-102* [15]. This suggests that cells are unable to progress through the S phase, likely due to the incapacity of the broken forks to re-start in a Rad51-dependent manner. The results are consistent with the idea that, in *rem* mutants, replication forks break at the damaged sites as a consequence of unfinished NER, channeling repair of the resulting DSBs to HR, which in turn becomes essential.

### Higher DNA affinity of TFIIH *in vivo* in ATP-binding groove mutants

As mentioned above, the three *rem* mutations so far identified localize at the ATP-binding groove of Rad3 (S4A Figure). Defects in Rad3 ATP binding cause an ATP-hydrolysis defect, which is known to increase the affinity of TFIIH for DNA [16]. This could explain the low efficiency of late NER steps in *rad3-102* cells [4], [15]. Therefore we wondered whether the different levels of damage capable of being repaired by HR in the three *rem* mutants would correlate with a gain of DNA affinity of the Rad3 mutant proteins. We hypothesized that any ATP-binding groove mutant should show a *rem-*like phenotype. Since ATP hydrolysis failure should also compromise the helicase activity, the exception would be the helicase-null mutants, in which the incision step of the NER reaction cannot occur and would therefore behave as NER-null mutants. This would be the case of *rad3-2*, also located in the ATP-binding groove (S4A Figure), which is unable to excise the damaged ssDNA [19].

**Figure 3:**
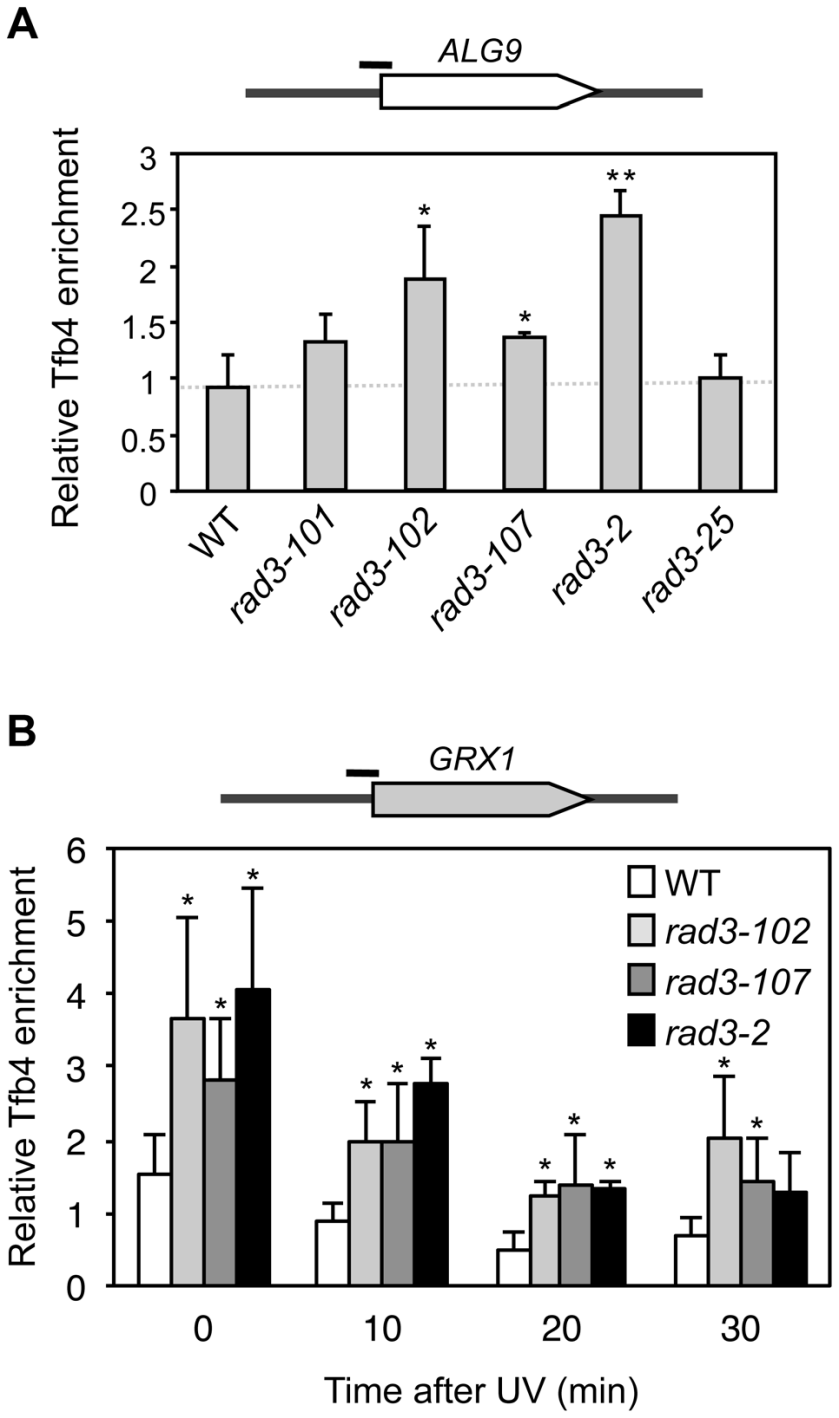
Analysis of TFIIH recruitment to promoters in *rad3* mutants. (**A**) Chromatin Immunoprecipitation (ChIP) analysis of Tfb4-TAP. Cells were grown in synthetic complete (SC) medium until the exponential phase. ChIP analysis was performed at the *ALG9* promoter and normalized with respect to the *MFA2* promoter in *MAT* **α** cells, which is constitutively repressed. (**B**) ChIP analysis of Tfb4-TAP after UV damage. Cells were grown in SC medium until the exponential phase, and then irradiated with 80 J/m^2^. Analysis of the different time-point samples was performed at the *GRX1* promoter and normalized with respect to the *MFA2* promoter in *MAT* **α** cells, which is constitutively repressed. The mean and the SD of triplicate assays of four independent experiments are depicted for each condition. *, p<0.05, **, p<0.01 (Student's t-test).

First, we asked whether mutations in the ATP-binding groove of Rad3, such as those of the *rem* strains studied here, could cause a gain in DNA affinity *in vivo*, independently of whether or not being masked by a helicase activity defect. For this we analyzed TFIIH retention at promoters, in which the helicase activity needed to open the DNA is provided by Rad25 and not by Rad3 [5]. We performed Tfb4-TAP chromatin immunoprecipitation (ChIP) in asynchronous cultures of the wild-type strain, the three *rem* mutants and the NER-null mutant *rad3-2*, plus the *rad3-25* mutant carrying a E548K amino acid change that maps at the DNA binding channel (S4A Figure), outside of the ATP-binding groove and that was therefore expected not to show a significant gain of ssDNA affinity. To minimize any possible effect caused by transcription, we determined TFIIH binding at the *ALG9* promoter, since *ALG9* is constitutively expressed at a constant rate independently of environmental and cellular conditions [20] and we verified that it was transcribed in all mutants to similar levels as the WT (S4B Figure). The ChIP analyses show that TFIIH is more abundant at the *ALG9* promoter in the three *rem* mutants, while in the *rad3-25* control recruitment was indistinguishable from the WT (Fig. 3A). The *rad3-2* NER-null mutant displayed the strongest promoter retention of all ATP-groove mutants, up to 3-fold the WT levels (Fig. 3A).

Then we analyzed promoter occupancy upon UV irradiation, as UV is known to drive TFIIH out of the promoters presumably to facilitate its action at NER sites [21]. We took samples at different time-points from asynchronous cultures during 30 minutes after UV irradiation of the WT and the strains showing a significantly enhanced promoter retention in Fig. 3A, namely *rad3-102, rad3-107* and *rad3-2* mutants, all of them mutated in the ATP-binding groove. We assayed TFIIH recruitment to the *GRX1* promoter, where we found the same relative increase as in the *ALG9* promoter under conditions of no irradiation. The high basal transcription levels of *GRX1* allow a better detection of falling off promoters upon UV treatment. Recruitment values for the control *rad3-25* were similar as those of the WT (S4C Figure). After an 80 J/m^2^ UV dose, the relative fall-off promoters experienced by TFIIH in each mutant background was similar (Fig. 3B), implying that the shut-off of transcription in response to UV to accomplish NER is intact. Nevertheless, a significant amount of TFIIH remained bound to the *GRX1* promoter in the *rad3-102, rad3-107* and *rad3-2* ATP-binding groove mutants up to 30 minutes, whereas almost most TFIIH was released from the promoter in WT cells (Fig. 3B). Altogether, these results indicate that mutants in the ATP-binding groove of Rad3 experience a gain in DNA affinity *in vivo* and, in particular, for promoters both with and without UV irradiation.

### Rad3 ATP-binding groove mutants generate highly diffusible TFIIH

**Figure 4:**
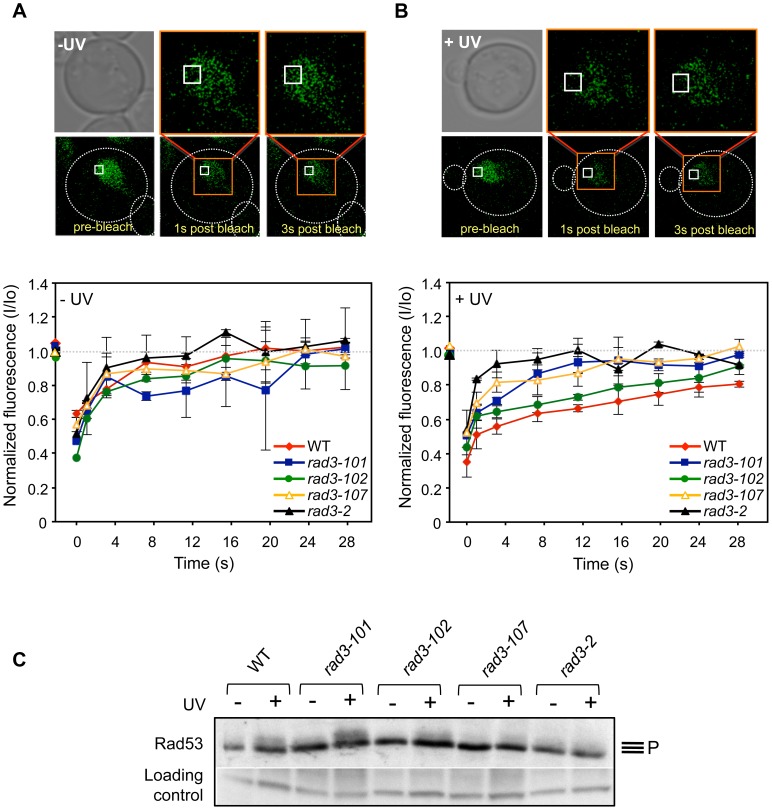
*In vivo* TFIIH complex dynamics in *rad3* mutants. (**A**) FRAP analysis of asynchronous non-irradiated cultures expressing yEGFP-Tfb4. Pictures show Tfb4-yEGFP fluorescence in the nucleus of WT cells. The white square indicates the bleached area. Curves show the evolution of the normalized fluorescence in the bleached area of the nucleus [fluorescence intensity at each time-point (I) between the initial value of fluorescence intensity (I_o_)]. Each represented value corresponds to the median value calculated from four consecutive time-points. Error bars indicate the SD of two independent experiments. (**B**) FRAP analysis of asynchronous 80 J/m^2^ UV-C-irradiated cultures expressing yEGFP-Tfb4. Details as in (A). (**C**) Total protein extracts of G1-synchronized cells that were either UV-irradiated (+) or not (-) with 100 J/m^2^ were probed for Rad53 phosphorylation. Control and Rad3 ATP-binding groove mutant cells are compared. Unspecific hybridization of the antibody is used as an inner loading control.

We had established the affinity for DNA *in vivo* of the mutant *Rad3* proteins by studying the residence of TFIIH at promoters. We then assayed globally the capacity of mutant TFIIH to bind to DNA at NER sites *in vivo* by using Fluorescence Recovery After Photo-bleaching (FRAP) using the tagged Tfb4-yEGFP protein. The rationale behind was that an impairment in the ATPase activity would lead to helicase activity defects and consequently to a defective performance of the protein during the repair reaction. Therefore, after UV, if *rad3* mutations prevented these activities of the protein, a bigger diffusing fraction should be observed for the mutants. We first confirmed that Tfb4-yEGFP behaves as a wild-type non-tagged Tfb4 by showing that the EGFP signal was detected all over the nuclei, that cells were as UV-resistant as WT cells and that an expected kinetics of fluorescence recovery was observed after photo-bleaching (Fig. 4A, and S5A and S5B Figure). In untreated WT cells, full recovery of fluorescence was observed in less than 12 seconds after bleaching (Fig. 4A and S5C Figure), consistent with the observation that most of the TFIIH complex within the nucleus is diffusible [22]. After UV irradiation a variable fraction of TFIIH is expected to move to the sites of DNA lesions to be engaged in their repair, leading to a low-diffusible fraction that reduces fluorescence recovery after bleaching. Accordingly, fluorescence recovery reached only up to 80% in cells treated with an 80 J/m^2^ UV dose (S5C Figure).

In spontaneous conditions, the TFIIH complex in ATP-groove mutants showed a similar dynamics to that of the WT (Fig. 4A). Nevertheless, after an 80 J/m^2^ UV dose, two observations could be made. First, fluorescence recovered completely in all mutants, indicating that the diffusing TFIIH fraction is bigger than in the WT (Fig. 4B). Second, the maximal recovery of fluorescence is achieved in shorter times in all mutants with respect to WT (Fig. 4B). Thus, it took only 8 seconds in *rad3-2* cells for maximal recovery, in sharp contrast to the 24 seconds needed for the WT. This is better appreciated when each time point is plotted relative to an identical normalized maximum (S5D Figure), in which it can be seen the difference in the slopes of the initial time points among the different strains. This result is consistent with the idea that a defect in the ATP-binding groove of Rad3 may hinder TFIIH ability to bind to repair sites, leading to a larger diffusion fraction.

### The *rem* phenotype recapitulates XP-D/CS-associated features

Accumulation of DNA breaks upon UV irradiation has been previously reported in XPD-deficient human XP8BR (XP-D/CS) cells [18] bearing a mutation in the ATP-binding groove of XPD. Interestingly, the equivalent mutant protein in *Sulfolobus acidocaldarius* exhibits an increased affinity for ssDNA [17]. Moreover, removal of the early-acting NER protein XPA suppressed the accumulation of DNA breaks [18]. Given that yeast *rem* mutants seem reminiscent of XP-D/CS cells, we investigated this putative parallelism.

In the first place, we analyzed the ability of *rem* mutants to activate the checkpoint in response to UV insults, since NER-deficient mutants do not accomplish damage processing, which in turn prevents checkpoint activation [23]. This feature is recapitulated in human cell lines defective for NER, including *XPD*-defective ones. Interestingly, cells from XP-D/CS patients, contrary to what was just mentioned, manage to activate the checkpoint [24]. Therefore, if XP-D/CS and *rem* mutations functionally relate, *rem* mutants should lead to checkpoint activation in response to UV. This prediction should apply to *rad3-101* and *rad3-102* mutants, who display an acute HR dependency, implying that initial NER steps are accomplished and therefore checkpoint activation can presumably occur. We synchronized cells in G1 and irradiated them with 100 J/m^2^ as previously described [23]. We monitored checkpoint activation in response to UV by following Rad53 phosphorylation. We could observe the expected phosphorylation of Rad53 in the WT strain (Fig. 4C). In agreement with the prediction, *rad3-101* and *rad3-102* displayed an even better, or slightly worse, respectively, Rad53 phosphorylation when compared with the WT (Fig. 4C). Very UV-sensitive mutants as *rad3-107* and *rad3-2*, related to poor damage processing, displayed negligible or absent checkpoint activation, respectively (Fig. 4C). As a whole, these data suggest a parallelism between *rem* and XP-D/CS-causing mutations and a molecular explanation for the reported checkpoint activation after UV described for XP-D/CS cells [24].

**Figure 5:**
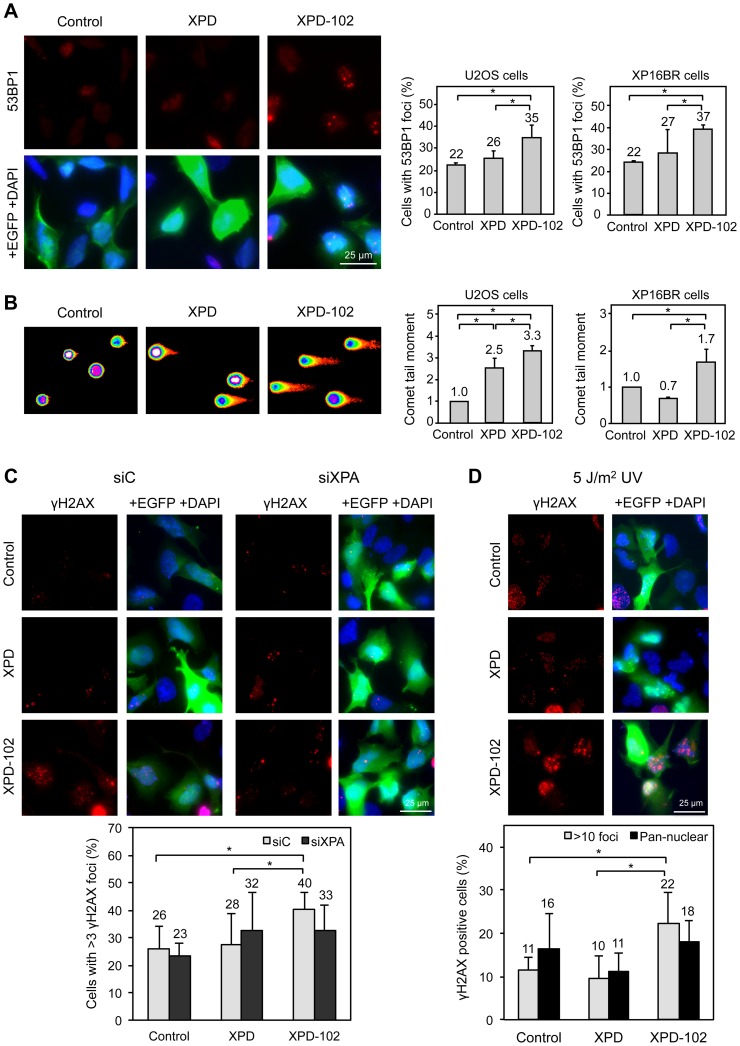
Evidences of DNA damage in XPD-102 human cells. (**A**) Immunofluorescence of U2OS cells and quantification of the number of U2OS and XP16BR cells containing 53BP1 foci after transfection with pIRES2-EGFP empty vector (Control), pIRES2-EGFP-*XPD* overexpressing the wild-type *XPD* allele (XPD) and pIRES2-EGFP-*XPD-102* overexpressing the *XPD*-*102 rem* allele (XPD-102). Expression of EGFP is used as a marker of transfection. Error bars indicate SD of three independent experiments. (**B**) Density map of the DNA single-cell electrophoresis from U2OS cells and quantification of the comet tail moments of U2OS and XP16BR cells transfected with the control, XPD and XPD-102 constructions. Details as in (A) (**C**) Immunofluorescence and quantification data of the number of U2OS Control, XPD and XPD-102 cells containing more than 3 γH2AX foci after transfection with siControl (siC) or siXPA. Error bars indicate the SD of four independent experiments. (**D**) Immunofluorescence and quantification of the number of U2OS control, XPD and XPD-102 cells containing either pan-nuclear staining or more than 10 discrete γH2AX foci 5 hours after irradiation with 5 J/m^2^ UV-C. Error bars indicate the SD of four independent experiments. *, p<0.05 (Mann-Whitney U test).

In the second place, we wanted to test whether the *rem* alleles had the same impact on human cells as in *S. cerevisiae*. For this, we first assayed whether the yeast *rad3-102* feature of accumulation of spontaneous DNA breaks was recreated in human cells. To achieve it, we overexpressed an *XPD* allele, *XPD-102* (*XPD-H659Y*), carrying the equivalent of the yeast semi-dominant *rad3-102* (*rad3-H661Y*) mutation (Fig. 5A). As a control we overexpressed the wild-type version of *XPD* from the same plasmid. mRNA levels of *XPD* and *XPD-102* increased 300-fold with respect to cells transfected with the empty vector after 24 hours of transfection, and this correlated with increased expression at the protein level (S6A Figure). As an additional control, we verified that *XPD-102* overexpression did not alter basic transcriptional patterns of the cell. Analysis by qPCR of levels of two relevant housekeeping mRNAs denoted no change in transcription between control and XPD-102-overexpressing cells (S6B Figure).

As DNA breaks accumulate in yeast *rad3-102* cells [15], we analyzed by immunofluorescence 53BP1 foci, known to accumulate early at sites of DSBs [25]. U2OS cells overexpressing wild-type *XPD* show a similar percentage of 53BP1 foci-containing cells as U2OS cells transfected with the empty plasmid (22% and 26%, respectively). However, the percentage of cells with 53BP1 foci was clearly increased in cells overexpressing *XPD-102* (35%) (Fig. 5A). For further evidence of the accumulation of DNA breaks, we directly analyzed the accumulation of broken DNA fragments using the single cell gel electrophoresis assay (*comet* assay) under alkaline conditions to detect both single-stranded and double-stranded DNA breaks. In these assays, XPD-102 cells showed a larger tail moment than XPD and control cells (Fig. 5B), which demonstrates that physical breaks are spontaneously produced in these cells. Conclusions were confirmed in U2OS cells by additional immunofluorescence analysis. While the percentage of U2OS cells containing γH2AX foci was similar in control and XPD-overexpressing cells, XPD-102 U2OS cells showed a significantly higher accumulation (Fig. 5C). We also validated this notion in HeLa cells, where the γH2AX signal increased 1.5 times after *XPD-102* overexpression with respect to *XPD*-overexpressing cells as determined by FACS (S6C Figure). As thus far experiments were performed overexpressing *XPD-102* in XPD^+/+^ cells, next we asked whether results were the same in primary XP16BR (XPD-R683W/R616P, XP-D) fibroblasts, in which one XPD allele is hypothesized to be null and the other one severely affected in its enzymatic activity [26]. In these primary fibroblasts, 53BP1 foci and comet tail moments were similar to those obtained in XPD^+/+^ U2OS cells, with a 1.7-fold increase above the control cells transfected either with the empty vector or the one overexpressing the wild-type allele of XPD (Fig. 5A and 5B). Altogether, these results imply that the basal level of damage recruiting TFIIH is sufficient to uncover the effects caused by the action of mutant *XPD-102*, which results in the production of breaks. We therefore conclude that the *XPD-102* mutation causes similar effects both in humans and yeast.

The breaks described to occur in XP-D/CS human XPD-G675R cells and in mouse *Xpd^G602^* cells are UV-induced, and in the latter case have been shown to depend on the NER-initiating factor XPA [18]. To prove the analogy between the *rem*-like mutation *XPD-102* and *XP-D/CS* cells, we first assessed whether XPA depletion by siRNA suppressed the breaks provoked by *XPD-102* without UV irradiation. XPA mRNA levels were similarly reduced in all cells after 96 hours of siRNA depletion, and this correlated with a decrease at the protein level (S6D Figure). XPA depletion did not have any suppressive effect on the number of γH2AX foci in the control or in cells overexpressing XPD (Fig. 5C). In contrast, the increase in the percentage of γH2AX foci-containing U2OS cells upon XPD-102 expression was reduced back to the level of cells overexpressing wild-type XPD and depleted of XPA (Fig. 5C). This argues in favor of the idea that, as in XP-D/CS cells, the accumulation of DNA breaks in XPD-102 cells depends on a functional NER pathway.

If our hypothesis is correct, the XPD-102-induced breaks should be exacerbated by UV treatment, as more substrates would become available for the mutant XPD-102 protein. Following the same approach as above, the percentage of γH2AX foci-containing U2OS cells was studied. UV light greatly increased γH2AX foci in all cells (compare Figs. 5C and 5D). We differentiated pan-nuclear staining, a response to UV irradiation dependent on initial attack of the damage by the NER components, from discrete foci formation, a readout of replication-associated DSBs, among others [27]. While pan-nuclear staining was virtually similar in all assayed cellular contexts, the increase of discrete γH2AX foci was significantly exacerbated for cells overexpressing XPD-102 in comparison with both controls (Fig. 5D).

Altogether, the results suggest a functional relationship between human XP-D/CS and yeast *rem* mutations, in particular *rad3-102*, all of them affecting the ATP-binding groove.

## Discussion

The TFIIH complex has central roles in cell physiology, as it is involved in both transcription and DNA repair. This explains that defects in any of its subunits lead to inherited diseases in humans of important clinical relevance. Understanding the molecular basis underlying the phenotypes shown by TFIIH patients bearing a combination of both *Xeroderma pigmentosum* and Cockayne Syndrome has been long pursued. The classical view invokes a defect in TCR that accounts for both the NER deficiency and the transcriptional defects [6]. We have previously discussed several similarities between XP-D/CS-causing mutations and the *S. cerevisiae rad3-102 rem* allele [28]. Nevertheless, there are also important differences between them. Thus, *rad3-102* cells are slightly sensitive to UV, while XP-D/CS cells are strongly UV-sensitive. Also, *rad3-102* cells do not show clear transcriptional defects, while XP-D/CS cells do. In this work, by addressing directly the molecular basis for the analogies and differences between several yeast *rem* mutants (*rad3-101, rad3-102* and the newly constructed *rad3-107*) and by characterizing the effects of XPD-102 in human cells, we show that *rem* mutants can be used as a model to understand XP-D/CS-associated molecular phenotypes.

We first show that the *rad3-102* ability to tolerate UV irradiation is not by itself a *rem* feature. On the contrary, there exists a gradient of UV sensitivity depending on the *rem* mutant analyzed (Fig. 1). *rad3-102* tolerance to UV is based on the fact that TFIIH is able to remain bound to the damage site longer, thus inhibiting gap filling and generating a DSB during replication. As a result HR becomes essential for survival [13]-[15]. This is also the case of *rad3-101* cells, which display UV resistance comparable to that of the WT strain and exhibit full dependence on HR factors Rad52 and the MRX complex for survival (Fig. 2B). Instead, *rad3-107* cells, which are extremely UV-sensitive, display only a synthetic growth defect in the absence of HR factors (Fig. 2B). A lower ability of TFIIH to load onto DNA and to remain bound, thus resulting in a lower frequency of NER reactions ending in DSBs, would explain the inverse correlation between the need of HR and UV sensitivity. Nevertheless, even if the amount of damage leading to DSBs is different in each *rem* mutant, replication forks could still break in most cases. Consistently, removal in the three *rem* mutants tested of both Pol32 and Rad51, which block HR-mediated fork restart in *S. cerevisiae*, lead to inviability (Fig. 2B) [15]. Therefore, UV-resistance is not an obligatory feature of *rem* mutants, their variable degree of UV sensitivity depending on the balance between the amount of NER-repairable damage that remains unprocessed and the amount that is directed into HR repair.

As *rem* mutations map to the ATP-binding groove of Rad3 (S4A Figure), and are fully conserved (S1 Figure), they provide a useful tool to understand the possible consequences of impairing ATP binding on NER processing. XPD alterations leading to ATP-binding defects may compromise helicase activity and, consequently, could influence helix opening and helicase translocation on the DNA [29]. Indeed, the mutants tested displayed levels of fluorescence recovery after UV superior to the WT, as determined by FRAP analysis (Fig. 4B), a measure indicating a problem to attach to damage sites. Additionally, difficulties in hydrolyzing ATP would also lead to a putative TFIIH gain in ssDNA affinity [16]. Since, during transcription, Rad25 helicase activity is in charge of promoter opening, while Rad3 is only needed for structural reasons [5], it was possible to use promoters to detect putative TFIIH retentions at DNA. Notably, at basal transcription levels, all ATP-binding groove mutants, including the *rad3-2* NER-null mutant, show higher levels of TFIIH recruitment to promoters than the WT strain or than a UV-sensitive strain [30] whose mutation did not map to the ATP-binding groove, a behavior maintained after UV irradiation (Fig. 3A and 3B). Nevertheless, the relative fall-off promoters upon UV irradiation experienced by all mutants tested was comparable to that of the WT, indicating a normal response to abrogate transcription and migrate to NER sites (Fig. 3B). These data support the notion that the TFIIH complexes in *rem* ATP-binding groove mutants display an enhanced affinity for DNA. Remarkably, all XPD mutations causative of XP-CS known to date are located in the ATP-binding groove (S4A Figure). Re-creation of these four mutations in *Sulfolobus acidocaldarius* XPD demonstrated a dramatic loss of both ATPase and helicase activities. Importantly, half of them exhibited an increased ssDNA affinity, a property not seen in any other category of XPD mutants [17].

Our first molecular observation to functionally link Rad3 *rem* mutations and XP-CS-causing XPD ones is the ability to activate the checkpoint in response to UV of both XPD-CS cells [24] and HR-dependent *rem* mutants (Fig. 4C). This is a remarkable parallelism since canonical *rad3* NER-deficient yeast and *XPD*-deficient human cells are unable to activate the checkpoint upon UV insult [23], [24]. Moreover, this provides an explanation for the mechanism leading to such checkpoint activation in XP-D/CS cells: partial processing of the lesion by accomplishment of the initial steps of NER, as it occurs in HR-dependent *rem* cells.

To search for additional molecular support for the *rem/*XP-D/CS link, we moved to human cells. Overexpression of an *XPD-102* (*XPD-H659Y*) mutation leads to DNA break accumulation, already in the absence of UV, as detected by single-cell electrophoresis as well as 53BP1 and γH2AX foci accumulation (Fig. 5 and S6C Figure) in three different human cell lines. This detection is possible since there exist substrates for NER under basal conditions that are processed by the defective XPD-102. Therefore, we are able to re-create in human cells this known feature of yeast *rad3-102*. Yet, if the notion that *rem* and XP-D/CS mutations are equivalent is correct, several predictions should prove right. First, such an accumulation of DNA breaks should be XPA-dependent. Indeed, depletion of XPA led to a reduction in the accumulation of DNA breaks to levels comparable to those caused by overexpression of the control XPD (Fig. 5C), as seen in XP-D/CS [18] and *rad3-102* cells, where early-acting factors such as Rad4 suppress this defect [14]. Second, the formation of breaks should be further increased by providing the cells with more substrates, namely by UV-irradiation. Again, a significant fraction of γH2AX foci accumulated specifically upon overexpression of XPD-102, while overexpression of the wild type allele of XPD did not even increase this value up to the level seen in the control itself (Fig. 5D). Last, even though XP-D/CS breaks were originally described as being transcription-dependent [18], this notion was recently dismissed using *Xpd^G602D^* mouse cells, which mimic an XP-D/CS mutation [31]. Indeed, it has been demonstrated that early steps of the DNA damage-signaling cascade, as those under study here, still occur even if transcription is inhibited, and that the action of the mutant protein has been exerted [31]. Altogether, the results support the view that *rem* alleles and human mutations causing XP-D/CS phenotype are functionally equivalent.

**Figure 6:**
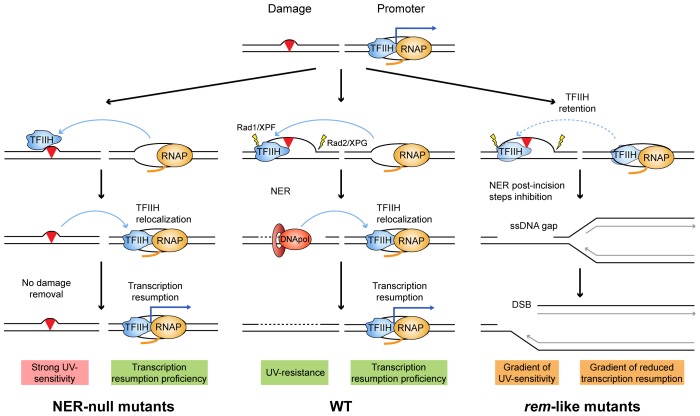
Model to explain the molecular defects occurring in XP-D/CS cells. Mutations in the ATP-binding groove of Rad3/XPD would compromise both its ATPase and its helicase activities. Depending on the mutation, both capacities would be compromised to different extents. A simplified scheme of each situation is illustrated by a paradigmatic yeast *rad3* mutation. The different envisioned stages of the NER engagement in repair and its impact on on-going transcription in each situation are depicted. The main molecular outcomes are highlighted in pink, green and orange.

The explanation underlying the molecular basis of XP-CS has remained elusive. The XP defect is explained by deficient NER, but the explanation for the CS phenotype, generally invoking a defect in TCR, is unclear. In our view, an integrated mechanism for DNA damage removal inability and transcription retardation can be proposed. In WT cells, TFIIH will promote NER and then resume transcription (Fig. 6, middle). Rad3/XPD null mutants will be unable to open DNA at a damaged site therefore not affecting transcription resumption (Fig. 6, left). However, in mutants of the ATP-binding groove (*rem*-like mutants, Fig. 6, right), there would co-exist an enhanced DNA affinity and a reduced DNA opening capacity. The increased DNA affinity would easily manifest at locations where TFIIH binding does not depend on Rad3/XPD, as it is the case of promoters. The enhanced affinity for other DNA sites, such as damaged DNA, nevertheless, would depend on the ability of TFIIH to open the DNA first. If the opening occurs, TFIIH could be engaged in NER but will stay bound to the damaged DNA for longer due to a gain in affinity for DNA. This may delay transcription resumption and also provoke replication fork collapse and breakage that would demand the intervention of the HR machinery. If this process were efficient, UV sensitivity would be mild. However, mutations in the ATP-binding groove may also have a strong impact, so that TFIIH may stay associated with promoters (Fig. 6, right, dashed lines). This model would be in agreement with the proposal that genomic DNA is cut *in trans* upon transfection of damaged plasmids into XP-CS cells [9]. In our view, therefore, the transcription impairment after DNA damage would occur in a TCR-independent manner. In further agreement with this view, recent data reveal that RNA recovery defects following UV in XP-CS cells are restricted to genes whose expression was shut-off specifically in response to UV, leaving damage-inducible genes unaffected, thus arguing against a general defect in TCR, which impairs all types of transcription [32]. Moreover, authors demonstrate a specific heterochromatinization happening at promoters that do not resume transcription [32], temptingly as a consequence of a too lasting period without TFIIH components coming back.

In conclusion, we propose that, as in the *rem* alleles, the XP features seen in XP-D/CS patients arise from the inability of their cells to initiate and/or accomplish a proficient NER reaction, whereas the CS features would be the consequence of increased levels of the repair conformation of TFIIH that may compromise resumption of transcription. Consistent with our model, it has recently been observed in *Xpd^G602D^* mice cells mimicking an XP-D/CS mutation that defective NER leads to the accumulation of long stretches of ssDNA upon UV, suggesting that the long-lasting aberrant NER intermediate extends the time of repair and therefore may inhibit transcription for long periods [31]. Subsequent accumulation of replication-mediated DNA breaks that demand an intact HR for its repair and replication restart would explain the high levels of genetic instability that characterizes both *rem* yeast and XP-CS human cells.

## Materials and methods

### Strains and plasmids

Strains used are described in S1 Table. The *rad3-107* (*rad3-E236G*) mutation was constructed by oligonucleotide directed mutagenesis using the primer 5'TTTTGATG(CGT)AGCGCACA3' and a plasmid pPF1 containing the *Bam*HI-*Sal*I fragment of *RAD3* cloned into pGEM7Zf (Promega Biotech, Madison, WI). The E236G mutation was confirmed by sequencing and was used to substitute the wild type *Bam*HI-*Sal*I fragment of pBM3 [14] to make pBM3-107. Plasmids pBM3-101 and pBM3-107, containing the *rad3-101* and *rad3-107* mutant alleles, were used to substitute the endogenous *RAD3* gene by the respective mutant alleles followed by the hygromycin resistance cassette to generate strains YREC57-41 and YREC57-45. Plasmid pKT127 [33] was used to construct Tfb4-yEGFP-based strains. All other single, double and triple mutants were obtained by backcrosses. Plasmid pWJ1344 carrying the *RAD52-YFP* construct has been previously described [34]. The *XPD* cDNA sequence was obtained by PCR from the RZPD/ImaGenes clone IRATp970F12108D (Berlin, Germany). The *XPD-102* allele containing the C1980T substitution was obtained by directed mutagenesis using two overlapping PCR primers (GGACCTGTAAGGCTCGAGATGAAGCTCAACGTGGA and GGGCCGCGTAGCGCATGGCATCGAAGGTAAGAAA for the 5′ site and ACTTTCTTACCTTCGATGCCATGCGCTACGCGGCCCAGT and GCGCTCGATACGGAATTCTCAGAGCTGCTGAGCAA for the 3′ site) sharing the mutation. Both cDNAs were cloned into the *Xho*I-*Eco*RI sites of the pIRES2-EGFP vector (Clontech, Palo Alto, CA, USA).

### Cell culture and transfection

U2OS and HeLa cells were cultured in DMEM (Gibco, NY) supplemented with 10% heat-inactivated fetal bovine serum (FBS). Primary human XP16BR fibroblasts (provided by Dr. A.R. Lehmann) were cultured in Eagle's minimum essential medium (Biowest) with 15% heat-inactivated FBS. All cells were maintained at 37°C and 5% CO_2_.

Short interfering RNAs (siRNA) used were on-target plus non-targeting pool (siC) and on-target plus smartpool human XPA (siXPA) (Dharmacon). Cells were transfected with plasmid (2 µg/ml) or siRNA (100 nM) using Lipofectamine 2000 (Invitrogen, Carlsbad, CA) or DharmaFECT 1 (Dharmacon) respectively, according to the manufacturer's instructions. Immunostaining and single-cell electrophoresis assays were performed 24 or 96 hours after the plasmid or siRNA transfection, respectively.

### Chromatin immunoprecipitation and quantitative PCR

Strains were cultured until 0.7_O.D.600nm_ in SC. A 50 mL sample was taken for the non-irradiated control experiments. The culture was centrifuged, resuspended in distilled, sterile water, and irradiated in plates using a 80 J/m^2^ UV dose. Cells were immediately resuspended in fresh medium, and 50 mL samples were taken every 10 min. Samples processing was performed as described [35]. IgG Sepharose (GE Healthcare) was incubated with samples over night to precipitate TAP-tagged Tfb4. The Wizard^R^ SV DNA clean-up system (Promega) was used for the last DNA purification step. Quantitative PCR (qPCR) was performed against the *ALG9* or *GRX1* loci promoters in *Mat*α cells. Normalization was done with values of amplification at the *MFA2* promoter, as described for *Mat*α cells in order to study TFIIH recruitment in the absence of transcription [36]. Damage of templates by UV irradiation did not impede proper qPCR amplification, since controls at sites of active transcription displayed high amplification signals, and *in vitro* repair of templates prior to qPCR as described [37] did not alter results. Primers used were: ALG9-fw: TGGCTCTTTTTTCACCCTGAA; ALG9-rv: TGGTTACCGCCTTGCAATTC; MFA2-fw: TGCATGTCAGAGGAAAAAGAACAAAG; MFA2-rv: CGGTGAACGACAGAAGAAGTGG; GRX1-fw: TCACGTGAATCAGGAGGCG; GRX1-rv: GGCGTTTCCAGATTGCGAT.

### Fluorescence recovery after photobleaching

Overnight Tfb4-yEGFP mid-log cultures, grown in YPAD, were either non-treated or 80 J/m^2^-UV-C-irradiated. FRAP was performed on a Leica TCS SP5 confocal microscope at room temperature. A 0.6 µm^2^ area into the nucleus was bleached at 50% laser intensity. Recovery of fluorescence into this area was monitored with 1 second intervals at 5% laser intensity. Images were acquired with a 488 nm laser. Fluorescence intensities were measured in bleached and unbleached areas with the MetaMorph v7.5.1.0. software. Normalized fluorescence in each point was calculated as follows: I_rel_  =  ((N_0_ - B_0_) × (I_t_ – B_t_))/((N_t_ – B_t_) × (I_0_ – B_0_)) where I_0_, N_0_ and B_0_ are the average intensity of the bleached region, an area in the same nucleus, or a randomly selected region outside of the cell during prebleach, respectively. I_t_, N_t_ and B_t_ are the average intensity of the bleached region, an area in the same nucleus, or a randomly selected region outside of the cell at each time point, respectively. At least 10 cells for each condition were analyzed.

### Immunofluorescence analysis

Cells were cultured on glass coverslips and transfected at 70-80% confluence. For UV irradiation, cells were washed with PBS, irradiated with a 5 J/m^2^ UV-C dose after PBS removal, further incubated and collected after 5 hours. Cells were fixed in 2% formaldehyde in phosphate-buffered saline buffer (PBS) for 20 min and permeabilized with 70% ethanol for 5 min at −20°C, 5 min at 4°C, and washed twice in PBS. After blocking with 3% bovine serum albumin (BSA) in PBS, the coverslips were incubated with rabbit polyclonal anti-53BP1 (NB100-304 Abyntec Biopharma) or mouse monoclonal anti-γH2AX (JBW301, 05-636 Millipore) primary antibodies (1∶500) diluted in 3% BSA in PBS for 1h at room temperature. Secondary goat anti-rabbit antibody conjugated with Alexa Fluor 568 or goat anti-mouse antibody conjugated with Alexa Fluor 546 (Invitrogen) in 3% BSA in PBS were used. Transfection efficiency was monitored by transfection into cells of pIRES2-EGFP, pIRES2-EGFP-XPD or pIRES2-EGFP-XPD-102 plasmids. DNA was stained with DAPI. Images were captured with a Leica DM6000 microscope equipped with a DFC390 camera (Leica). Data acquisition was performed with LAS AF (Leica) and analyzed with the MetaMorph v7.5.1.0. software. More than 100 cells from each experiment were analyzed.

### Single-cell gel electrophoresis

Comet assay was performed using a commercial kit (Trevigen, Gaithersburg, MD, USA) following the manufacturer's protocol. Images were acquired as described above and analyzed with the Comet-score software (version 1.5). More than 100 cells from each experiment were scored.

### Reverse transcription qPCR analysis

cDNA was synthesized from total RNA extracted using RNeasy Mini Kit (Qiagen) (1 µg) by reverse transcription using Super-Script TM First strand synthesis for RT-PCR (Invitrogen, Carlsbad, CA) and random primers. RT-qPCR was performed with SYBR qPCR Mix and analyzed on an ABI Prism 7000 (Applied Biosystems, Carlsbad, CA). mRNA expression of the indicated genes were normalized with mRNA expression of the *HPRT* housekeeping gene. For the analysis of *DHFR* and *GAPDH* mRNA expression, data were normalized to the 18S RNA levels. Primers used were AAAGTGTCCGAGGGAATCGA and GGGACGCCAAACATGATGA for *XPD*, GAACCACTTTGATTTGCCAACTT and TTGCCTCTGTTTTGGTTATAAGCTT for *XPA*, GGACTAATTATGGACAGGACTG and TCCAGCAGGTCAGCAAAGAA for *HPRT*, TGCCACCAACTATCCAGACCA and CCTGGTTCTCCATTCCTGAGA for *DHFR*, CGACCACTTTGTCAAGCTCA and TACTCCTTGGAGGCCATGTG for *GAPDH*, and ATTCGAACGTCTGCCCTATCA and GTCACCCGTGGTCACCATG for 18S.

### Miscellanea

γH2AX signal in HeLa cells by FACS was measured using a commercial kit (FlowCellect Multi-Color DNA Damage Response Kit, Millipore, Germany) following the manufacturer's protocol. The methods for the analyses of yeast UV survival curves, cell cycle profiles, Pulsed-Field Gel Electrophoresis and spontaneous and UV-induced Rad52 foci have been previously described in detail [15]. Cell treatment, protein extraction and Western Blotting for Rad53 activation has been performed strictly as previously described [23]. Rad53 antibody used was previously described [38]. For XPD and XPA Western blots, 25 µg of total amount of protein, extracted from U2OS cells, were used. Antibodies XPD (abcam ab54676, 1∶5000), XPA (abcam ab2352, 1∶1000) and β-Actin (abcam ab8226, 1∶5000) diluted in TBS-Tween 0.1% with 5% milk were incubated overnight at 4°C.

## Supporting Information

S1 Figure Alignment of *Homo sapiens* XPD and *Saccharomyces cerevisiae* Rad3 protein sequences. Relevant residues (mutations) for this work are highlighted in blue for human and in red for yeast. Alignment was performed with ClustalW.
(TIF)

S2 Figure FACS profiles from WT, *rad3-101, rad3-102* and *rad3-107* cells. FACS from WT, *rad3-101, rad3-102* and *rad3-107* cells synchronized in G1 with α-factor, UV-irradiated with 20 J/m^2^ and released after 2 h.
(TIF)

S3 Figure UV sensitivity of *rad3-101 mre11Δ* and *rad3-107 mre11Δ* double mutants. Serial 10-fold dilutions of WT, *mre11Δ*, *rad3-101*, *rad3-107* and the corresponding double mutants grown in YPAD and UV irradiated with 5 J/m^2^.
(TIF)

S4 Figure Impact of ATP-binding groove mutations in XPD/Rad3. (**A**) 3D view of *Sulfolobus acidocaldarius* XPD. Equivalent residues to those relevant for this work have been selected in three different panels. Left panel corresponds to mutations causative of XP-CS in humans. HsG675, G47, R666 and G602 are indicated as SaC523, G34, R514 and G447. Middle panel corresponds to *rem* mutations in *S. cerevisiae*. ScA237 (*rad3-101*), H661 (*rad3-102*), E236 (*rad3-107*) and G461 (*rad3-2*) are indicated as SaA182, T507, E181 and G323. Right panel corresponds to *S. cerevisiae rad3-25* mutation, located in the DNA channel of XPD. ScE548 is indicated as SaD407. Protein Data Bank ID code is 3CRV. Image was processed with RasMol. (**B**) Northern hybridization of *ALG9* of mid-log phase cultures. (**C**) ChIP analysis of Tfb4-TAP in WT and *rad3-25* cells growing in synthetic complete medium until the exponential phase. The mean and the SD of triplicate assays of two independent experiments are depicted for each condition.
(TIF)

S5 Figure Analysis of Tfb4-yEGFP cells functions. (**A**) Microscopy images of WT and *Tfb4-yEGFP* cells showing the Tfb4-yEGFP signal in the nucleus. PH: phase contrast light image. (**B**) Serial dilutions of WT and *Tfb4-yEGFP* cells plated onto YPAD medium and UV-irradiated. (**C**) FRAP of asynchronous WT cultures of untreated and 80 J/m^2^ UV-C-irradiated cells. Curves show the recovery of the fluorescence at the bleached area. Each value corresponds to the median calculated from four consecutive time points. Error bars indicate the SD of three independent experiments. (**D**) FRAP curves of 80 J/m^2^ UV-C-irradiated cultures. Each value was normalized with respect to the maximal value of their curve, which was considered 1. Details as in (C).
(TIF)

S6 Figure mRNA and protein levels after transfection and DNA damage detection in XPD and XPD-102 cells. (**A**) Western of XPD in U2OS cells 24 h after the transfection with pIRES2-EGFP (Control), pIRES2-EGFP-*XPD* (XPD) and pIRES2-EGFP-*XPD-102* (XPD-102). β-Actin was used as a loading control. Relative quantification (RQ) data of the amount of *XPD* mRNA in U2OS and XP16BR cells 24 h after transfection as determined by qPCR. Error bars represent the minimum and maximum RQ of triplicate assays as determined using the comparative C_T_ method. (**B**) Relative quantification data of the amount of *DHFR* and *GAPDH* mRNA in U2OS cells 24 h after transfection as determined by qPCR. Error bars indicate the SD of five independent experiments. *, p<0.05 (Mann-Whitney U test). (**C**) Percentage of HeLa cells transfected with pIRES2-EGFP (Control), pIRES2-EGFP-*XPD* (XPD) and pIRES2-EGFP-*XPD-102* (XPD-102) with γH2AX signal, as detected by FACS. (**D**) Western of XPA and XPD in U2OS cells 24 h and 96 h after the plasmid and siRNA transfection, respectively. β-Actin was used as a loading control. Relative quantification data of the amount of *XPA* mRNA in U2OS cells 96 h after siRNA transfection as determined by qPCR. Details as in (A).
(TIF)

S1 Table *S. cerevisiae* strains used in this study.
(TIF)
