# Supplementary figures and images for: The rem Mutations in the ATP-Binding Groove of the Rad3/XPD Helicase Lead to Xeroderma pigmentosum-Cockayne Syndrome-Like Phenotypes
Source: PLoS Genet. 2014 Dec 11;10(12):e1004859. doi: 10.1371/journal.pgen.1004859 (PMC4263401; doi:10.1371/journal.pgen.1004859)

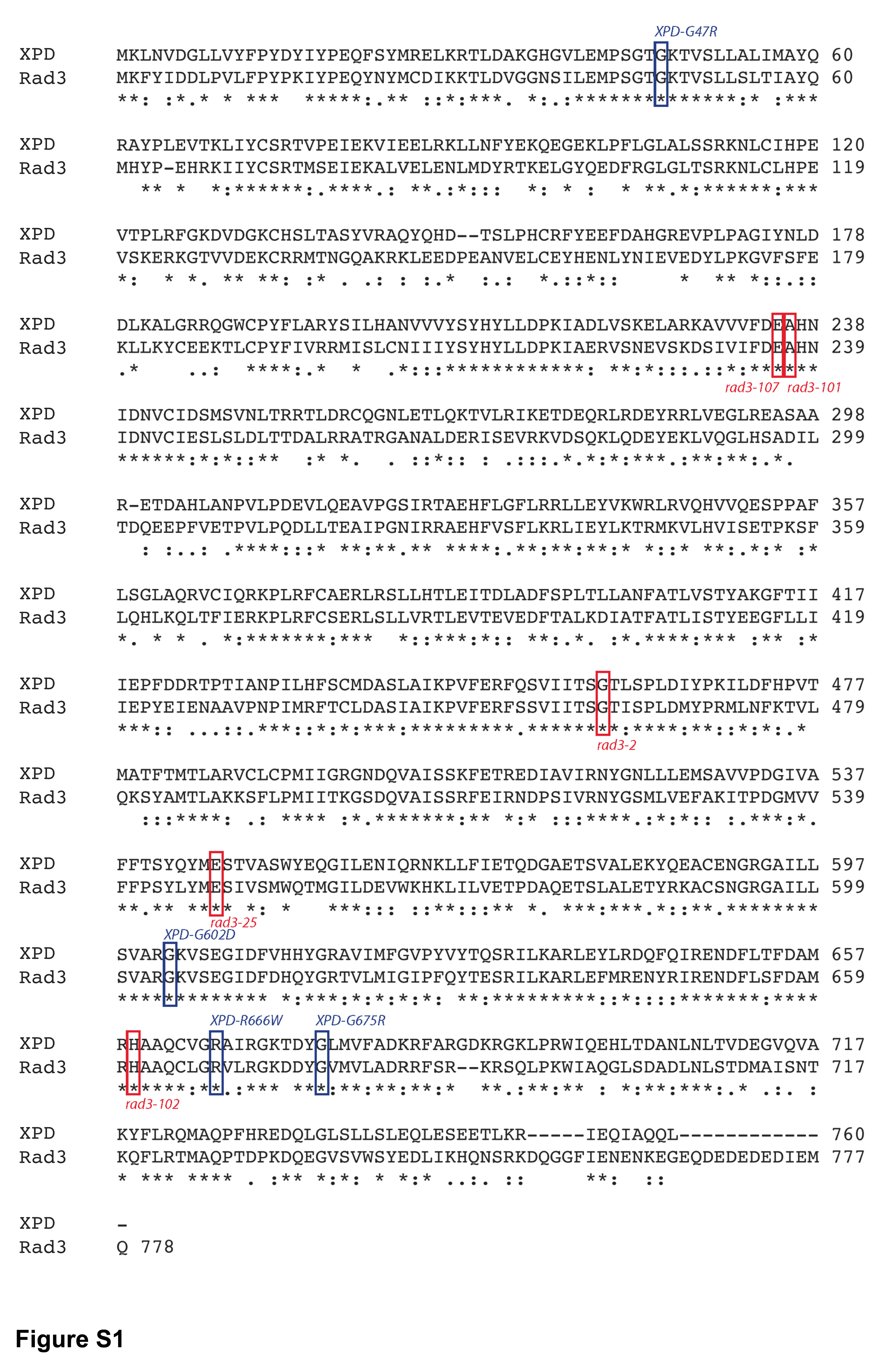

Supplement: S1 Figure — Alignment of Homo sapiens XPD and Saccharomyces cerevisiae Rad3 protein sequences. Relevant residues (mutations) for this work are highlighted in blue for human and in red for yeast. Alignment was performed with ClustalW. (TIF) [file pgen.1004859.s001.tif]

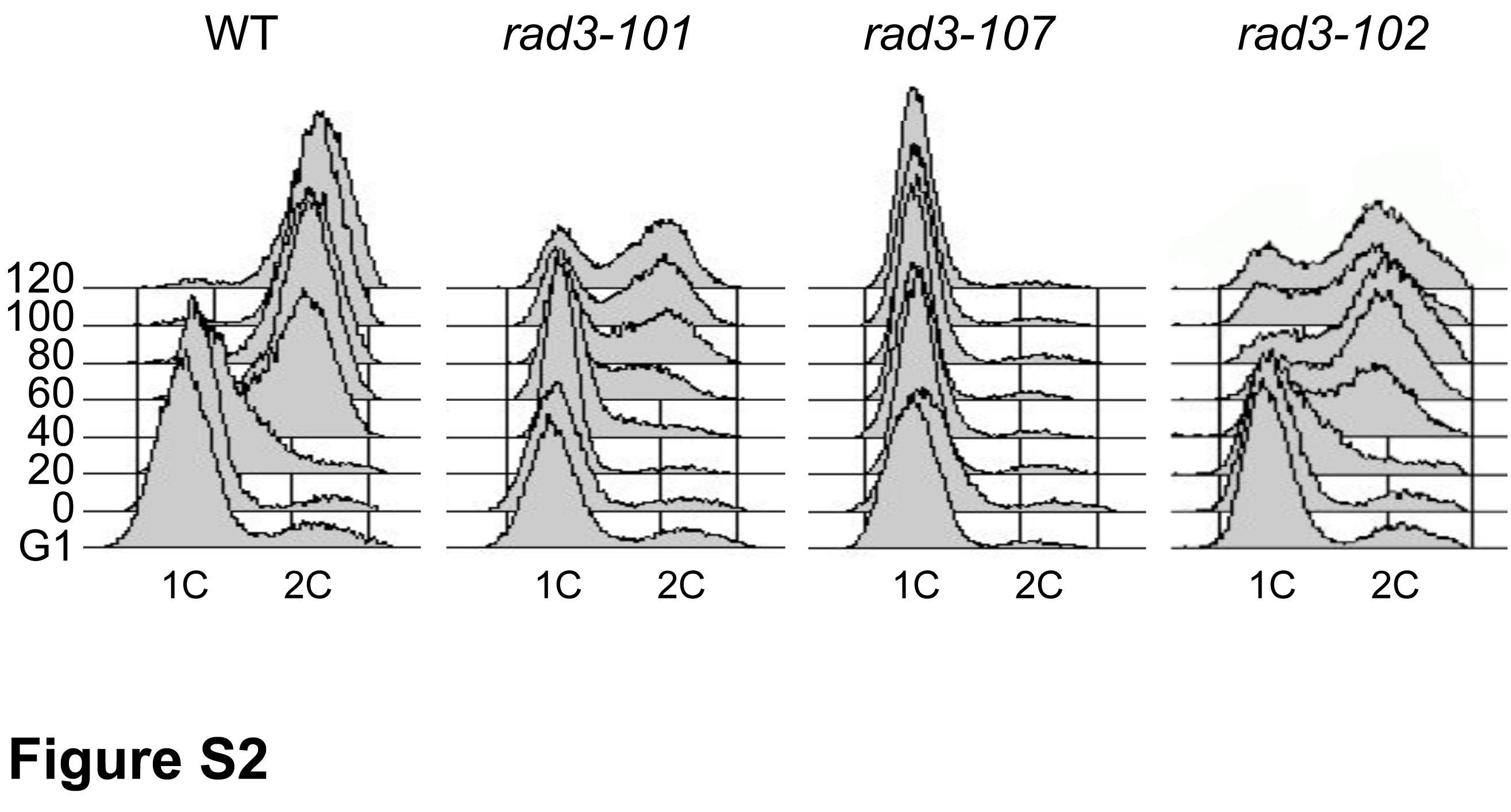

Supplement: S2 Figure — FACS profiles from WT, rad3-101, rad3-102 and rad3-107 cells. FACS from WT, rad3-101, rad3-102 and rad3-107 cells synchronized in G1 with α-factor, UV-irradiated with 20 J/m2 and released after 2 h. (TIF) [file pgen.1004859.s002.tif]

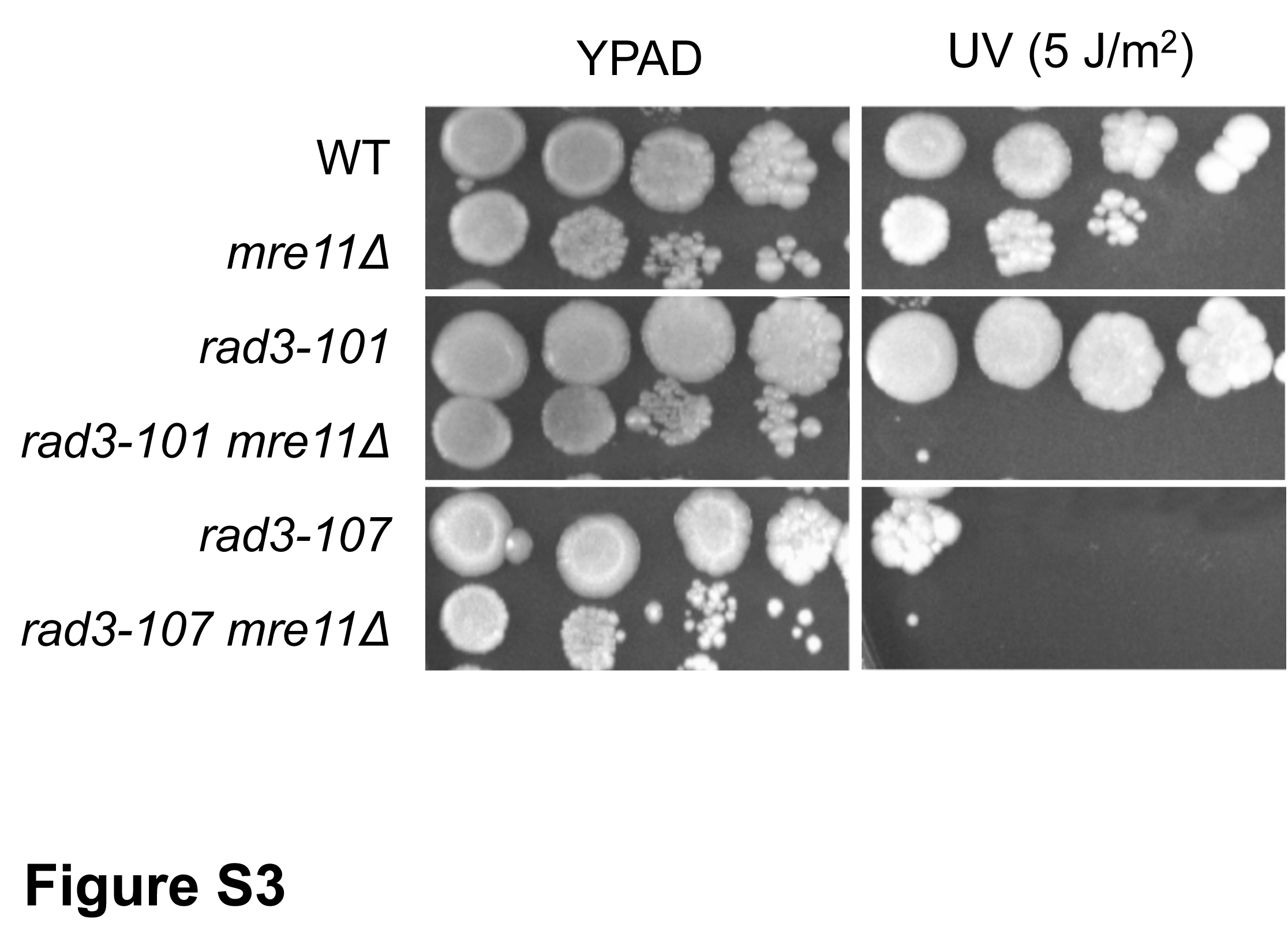

Supplement: S3 Figure — UV sensitivity of rad3-101 mre11Δ and rad3-107 mre11Δ double mutants. Serial 10-fold dilutions of WT, mre11Δ, rad3-101, rad3-107 and the corresponding double mutants grown in YPAD and UV irradiated with 5 J/m2. (TIF) [file pgen.1004859.s003.tif]

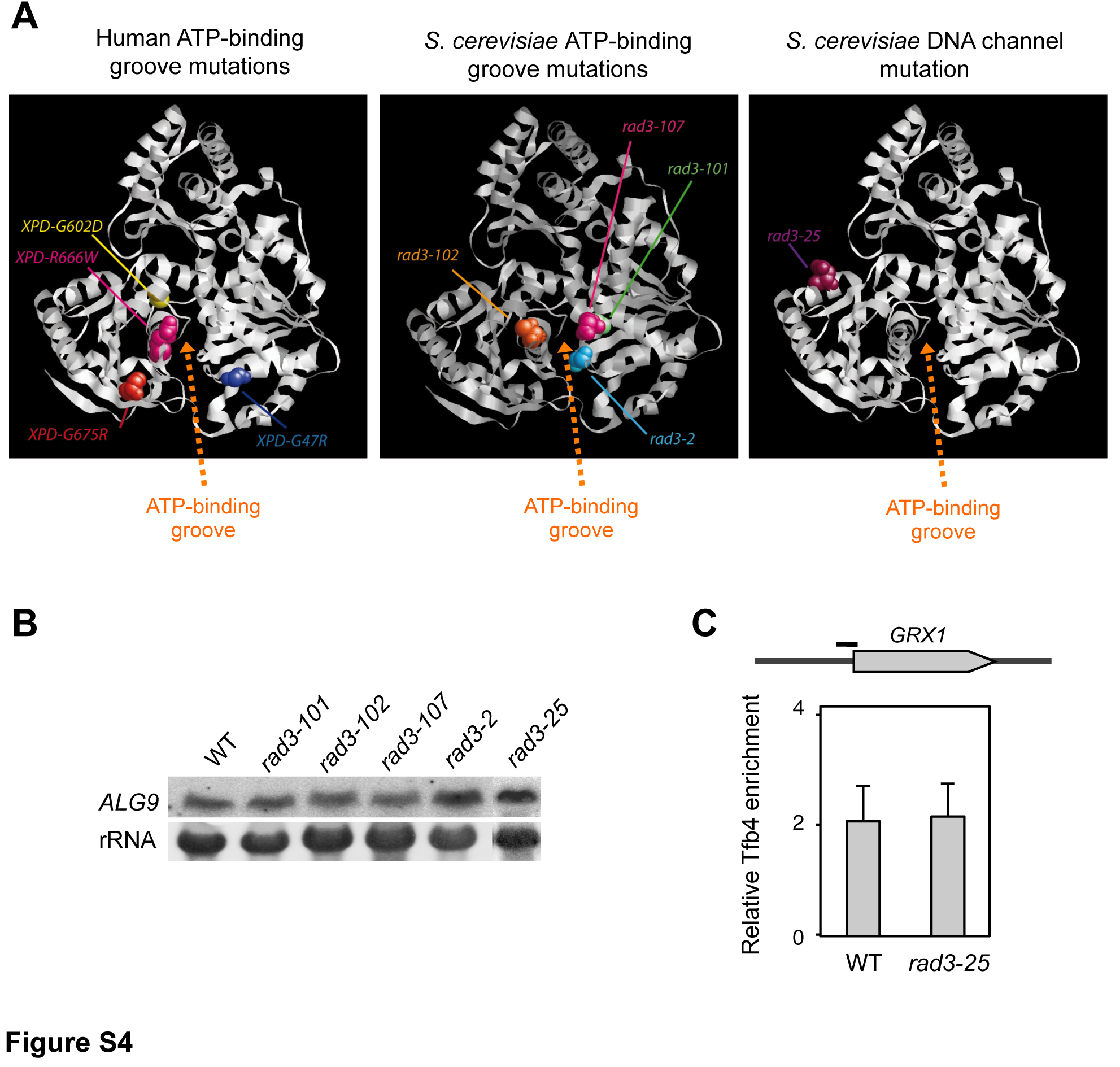

Supplement: S4 Figure — Impact of ATP-binding groove mutations in XPD/Rad3. (A) 3D view of Sulfolobus acidocaldarius XPD. Equivalent residues to those relevant for this work have been selected in three different panels. Left panel corresponds to mutations causative of XP-CS in humans. HsG675, G47, R666 and G602 are indicated as SaC523, G34, R514 and G447. Middle panel corresponds to rem mutations in S. cerevisiae. ScA237 (rad3-101), H661 (rad3-102), E236 (rad3-107) and G461 (rad3-2) are indicated as SaA182, T507, E181 and G323. Right panel corresponds to S. cerevisiae rad3-25 mutation, located in the DNA channel of XPD. ScE548 is indicated as SaD407. Protein Data Bank ID code is 3CRV. Image was processed with RasMol. (B) Northern hybridization of ALG9 of mid-log phase cultures. (C) ChIP analysis of Tfb4-TAP in WT and rad3-25 cells growing in synthetic complete medium until the exponential phase. The mean and the SD of triplicate assays of two independent experiments are depicted for each condition. (TIF) [file pgen.1004859.s004.tif]

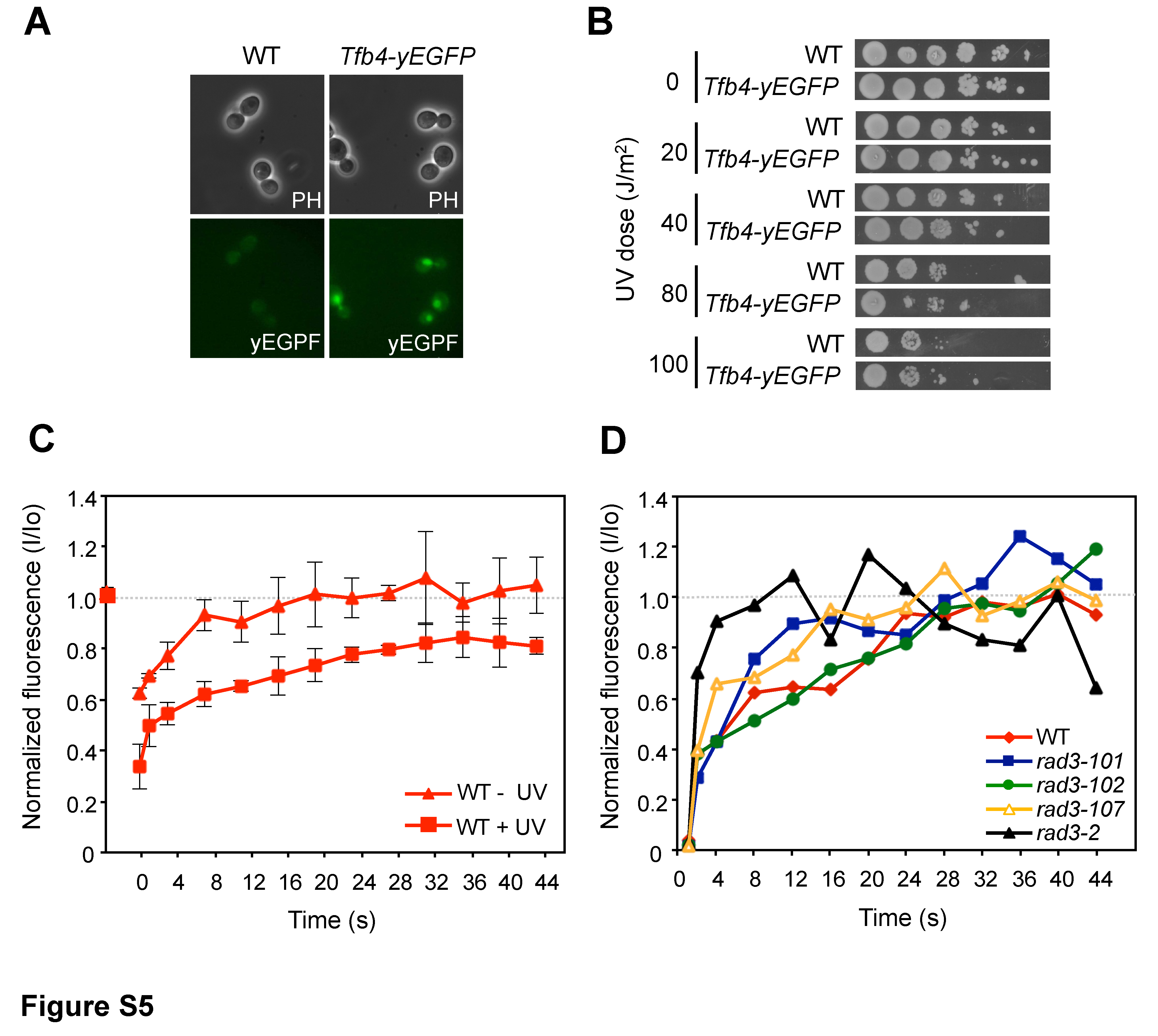

Supplement: S5 Figure — Analysis of Tfb4-yEGFP cells functions. (A) Microscopy images of WT and Tfb4-yEGFP cells showing the Tfb4-yEGFP signal in the nucleus. PH: phase contrast light image. (B) Serial dilutions of WT and Tfb4-yEGFP cells plated onto YPAD medium and UV-irradiated. (C) FRAP of asynchronous WT cultures of untreated and 80 J/m2 UV-C-irradiated cells. Curves show the recovery of the fluorescence at the bleached area. Each value corresponds to the median calculated from four consecutive time points. Error bars indicate the SD of three independent experiments. (D) FRAP curves of 80 J/m2 UV-C-irradiated cultures. Each value was normalized with respect to the maximal value of their curve, which was considered 1. Details as in (C). (TIF) [file pgen.1004859.s005.tif]

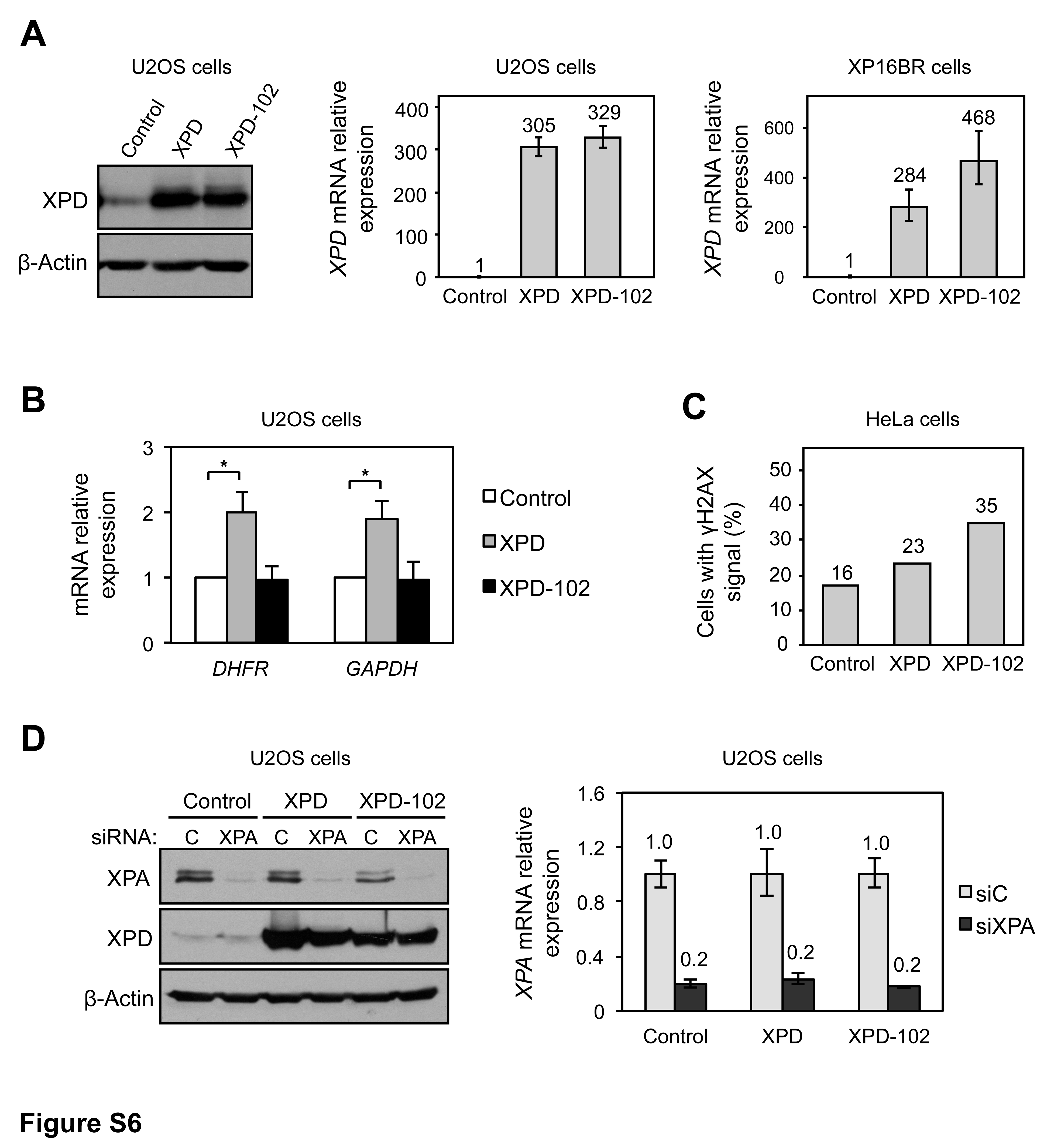

Supplement: S6 Figure — mRNA and protein levels after transfection and DNA damage detection in XPD and XPD-102 cells. (A) Western of XPD in U2OS cells 24 h after the transfection with pIRES2-EGFP (Control), pIRES2-EGFP-XPD (XPD) and pIRES2-EGFP-XPD-102 (XPD-102). β-Actin was used as a loading control. Relative quantification (RQ) data of the amount of XPD mRNA in U2OS and XP16BR cells 24 h after transfection as determined by qPCR. Error bars represent the minimum and maximum RQ of triplicate assays as determined using the comparative CT method. (B) Relative quantification data of the amount of DHFR and GAPDH mRNA in U2OS cells 24 h after transfection as determined by qPCR. Error bars indicate the SD of five independent experiments. *, p<0.05 (Mann-Whitney U test). (C) Percentage of HeLa cells transfected with pIRES2-EGFP (Control), pIRES2-EGFP-XPD (XPD) and pIRES2-EGFP-XPD-102 (XPD-102) with γH2AX signal, as detected by FACS. (D) Western of XPA and XPD in U2OS cells 24 h and 96 h after the plasmid and siRNA transfection, respectively. β-Actin was used as a loading control. Relative quantification data of the amount of XPA mRNA in U2OS cells 96 h after siRNA transfection as determined by qPCR. Details as in (A). (TIF) [file pgen.1004859.s006.tif]

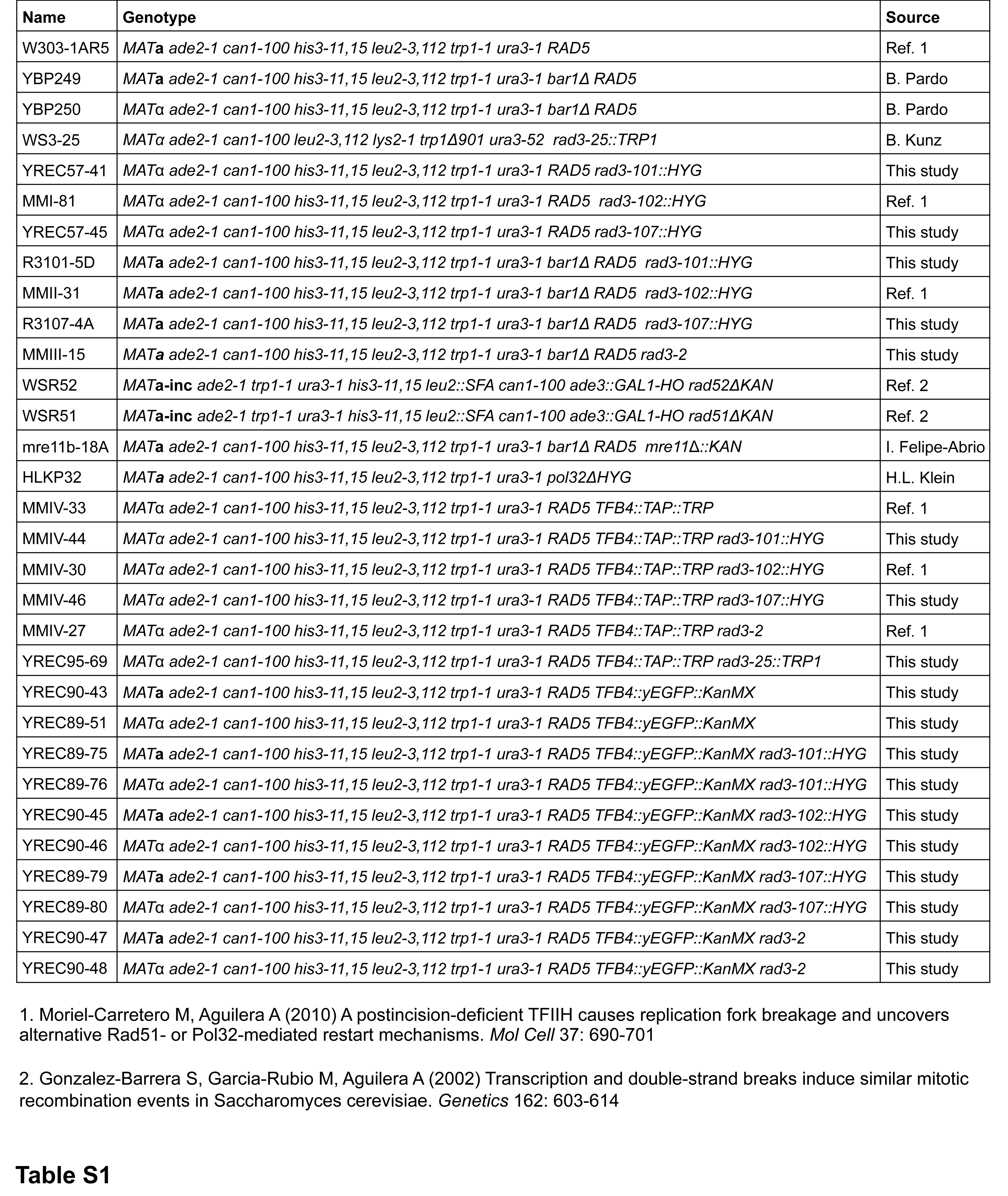

Supplement: S1 Table — S. cerevisiae strains used in this study. (TIF) [file pgen.1004859.s007.tif]
